# Carotenoids Database: structures, chemical fingerprints and distribution among organisms

**DOI:** 10.1093/database/bax004

**Published:** 2017-02-26

**Authors:** Junko Yabuzaki

**Affiliations:** a Center for Information Biology, National Institute of Genetics, Yata 1111, Mishima, Shizuoka 411-8540, Japan

## Abstract

To promote understanding of how organisms are related via carotenoids, either evolutionarily or symbiotically, or in food chains through natural histories, we built the Carotenoids Database. This provides chemical information on 1117 natural carotenoids with 683 source organisms. For extracting organisms closely related through the biosynthesis of carotenoids, we offer a new similarity search system ‘Search similar carotenoids’ using our original chemical fingerprint ‘Carotenoid DB Chemical Fingerprints’. These Carotenoid DB Chemical Fingerprints describe the chemical substructure and the modification details based upon International Union of Pure and Applied Chemistry (IUPAC) semi-systematic names of the carotenoids. The fingerprints also allow (i) easier prediction of six biological functions of carotenoids: provitamin A, membrane stabilizers, odorous substances, allelochemicals, antiproliferative activity and reverse MDR activity against cancer cells, (ii) easier classification of carotenoid structures, (iii) partial and exact structure searching and (iv) easier extraction of structural isomers and stereoisomers. We believe this to be the first attempt to establish fingerprints using the IUPAC semi-systematic names. For extracting close profiled organisms, we provide a new tool ‘Search similar profiled organisms’. Our current statistics show some insights into natural history: carotenoids seem to have been spread largely by bacteria, as they produce C30, C40, C45 and C50 carotenoids, with the widest range of end groups, and they share a small portion of C40 carotenoids with eukaryotes. Archaea share an even smaller portion with eukaryotes. Eukaryotes then have evolved a considerable variety of C40 carotenoids. Considering carotenoids, eukaryotes seem more closely related to bacteria than to archaea aside from 16S rRNA lineage analysis.

**Database URL**: http://carotenoiddb.jp

## Introduction

**Figure 1.**
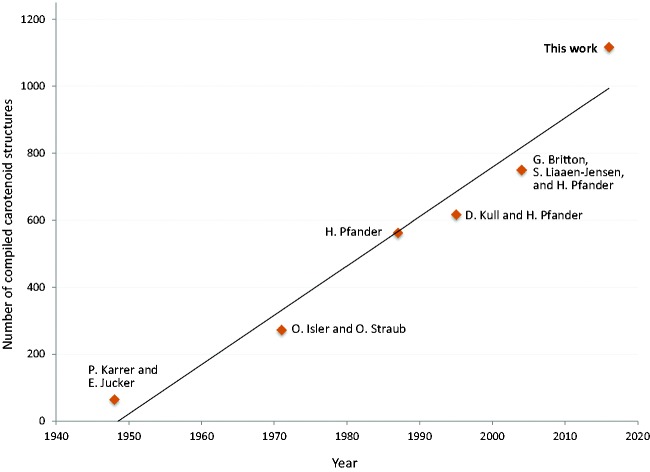
Growth curve of compiled carotenoid structures.

Carotenoids have been investigated due to the importance of their diverse biological functions, since the beginning of the 19th century (1). Investigations of their molecular structures were triggered by the successful determination of the structures of lycopene and β-carotene by Paul Karrer *et al.* in 1930 (2). The number of compiled carotenoid structures can be estimated to have risen almost linearly with time since 1948, that is, at about 15 structures per year on average (see Figure 1). The growth curve shows no saturation yet, implying the existence of many carotenoids yet to be identified. According to Carotenoids Handbook (3), about 30 well-assigned natural carotenoids plus another 30–40 non-fully characterized carotenoids were compiled by Paul Karrer and Ernst Jucker in 1948. In 1971, 273 carotenoids were compiled by Otto Isler *et al.* in the book ‘Carotenoids’ (4) and by Otto Straub in the book ‘Key to Carotenoids’ (5). In 1987, 563 carotenoids were compiled in the ‘Key to Carotenoids, second edition’ by Hanspeter Pfander (6). In 1995, D. Kull and H. Pfander added 54 new carotenoids as ‘Appendix’ (7).

In 2004, 750 carotenoids were compiled by George Britton, Synnφve Liaaen-Jensen and Hanspeter Pfander in the ‘Carotenoids Handbook’ (3).

In the course of evolution, carotenoids have been developed to perform diverse functions, probably starting with photosynthetic and photoprotective pigments and later sources of color, odor and taste. All biological functions investigated here are listed at http://carotenoiddb.jp/Biological_activity/biological_activities_list.html.

Organisms are sometimes related via carotenoids symbiotically as in the case of *Arbuscular mycorrhizae* accumulating the apocarotenoid mycorradicin in plant-roots during colonization (8). Diatoms produce the feeding deterrent apocarotenoids apo-fucoxanthinals and apo-fucoxanthinones against copepods, which may significantly influence food chains (9, 10).

For deeper understanding of the world of carotenoids—how organisms are related via carotenoids, either evolutionarily, or symbiotically, or in food chains through natural histories, and how carotenoids have been evolved with biological functions, we compiled 1117 structures and their distribution among organisms using the latest available original papers. We made these data accessible via the Internet at ‘http://carotenoiddb.jp’.

Aiming to extract organisms closely related through the biosynthesis of carotenoids, we developed a precise similarity search system exploiting the ‘Carotenoid DB Chemical Fingerprints’ from the IUPAC semi-systematic names. IUPAC semi-systematic names are very well defined to fully represent the chemical structures (11).

The Carotenoid DB Chemical Fingerprints describe the chemical substructure and modification details with modified carbon-numbering; for example, ‘3-OH, 3′-OH, 4 = O, 4′=O, beta,beta’ for astaxanthin. The carbon-numbering and the naming system follow the Nomenclature of Carotenoids approved by the IUPAC and International Union of Biochemistry (IUB) commissions (11). Our fingerprints are unique in including positional information. 

Consequently, precise similarity searching has been achieved by a simple scoring method.

The chemical fingerprints also allow (i) easier prediction of biological functions of carotenoids, (ii) easier classification of carotenoid structures, (iii) partial structure searching by simple string searches ‘psi,psi 4-apo 4-al’ for instance, from the search box ‘http://carotenoiddb.jp/search.cgi’ and (iv) easier extraction of structural isomers and stereoisomers.

It is worth noting that this is the first attempt, to our knowledge, to establish fingerprints from IUPAC semi-systematic names.

## Carotenoids Database information

The Carotenoids Database provides carotenoid chemical information, distribution among source organisms, and biological functions of carotenoids. A list of all the carotenoids compiled here is available at ‘http://carotenoiddb.jp/Entries/list1.html’. Information on each carotenoid is described in each entry. All the entries can be searched with a free word retrieval system at ‘http://carotenoiddb.jp/search.cgi’. Information in each entry can be categorized in six types, namely, (i) name information, (ii) hierarchical classification, (iii) structural information, (iv) biological functions, (v) chemical properties and (vi) source organisms. The details are described in Table 1.

**Table 1.** The data content of carotenoid entries (December 2016 release)

Field	Data content
ENTRY	Accession number which begins with CA
HIERARCHICAL CLASSIFICATION	Classification by the number of carbon atoms, end groups and chemical modification patterns
NAME	Trivial name of the carotenoid
IUPAC NAME	Systematic-name abide by nomenclature of carotenoids approved by the IUPAC and the IUPAC-IUB Commission
FORMULA	Chemical formula calculated by Open Babel
MOLECULAR WEIGHT	Molecular weight calculated with Standard Atomic Weights 2015 which are defined by the Chemical Society of Japan
CHEMICAL STRUCTURE	PNG file and Mol file of our own handwriting carotenoid structure
CHEMICAL FINGERPRINTS	Carotenoid DB Chemical Fingerprints investigated in this work
ISOMERS	Accession numbers of constitutional isomers, and stereoisomers which include *cis*/*trans* isomers, conformers, and enantiomers
BIOLOGICAL FUNCTIONS AND PROPERTIES	Photosynthetic pigment, photoprotective agent, provitamin A, antioxidant, anticarcinogenic activity, colour, etc.
InChI	The IUPAC International Chemical Identifier converted by Open Babel
InChIKey	Fixed-length (27-character) condensed digital representation of an InChI converted by Open Babel
Canonical SMILES	Canonical Simplified Molecular Input Line Entry System converted by Open Babel
XLogP	Partition coefficient calculated by PaDEL-Descriptor
HYDROGEN BOND DONORS	Number of hydrogen bond donors (using Lipinski's definition: Any OH or NH. Each available hydrogen atom is counted as onehydrogen bond donor) calculated by PaDEL-Descriptor
HYDROGEN BOND ACCEPTORS	Number of hydrogen bond acceptors (using Lipinski's definition: any nitrogen; any oxygen) calculated by PaDEL-Descriptor
LIPINSKI FAILURES	Number failures of the Lipinski's Rule Of 5 calculated by PaDEL-Descriptor
COMPLEXITY OF MOLECULE	Complexity of a molecule calculated by PaDEL-Descriptor
NUMBER OF HEAVY ATOMS	Number of heavy atoms (i.e. not hydrogen) calculated by PaDEL-Descriptor
TOPOLOGICAL POLAR SURFACE AREA	Sum of solvent accessible surface areas of atoms with absolute value of partial charges greater than or equal to 0.2 calculated by PaDEL-Descriptor
SOURCE ORGANISMS	Scientific names of source organisms obtained from the latest available papers
REFERENCES	References of original papers
CAS	Chemical Abstract Service number
LINKS TO OTHER DB	Links to KEGG COMPOUND, KNApSAcK, Lipidbank and ProCarDB

The carotenoid profile of one source organism is described in each organism entry. A list of all organisms in the Carotenoids Database is available at ‘http://carotenoiddb.jp/ORGANISMS/all_org.html’. Organism entries are also searchable with a free word retrieval system at ‘http://carotenoiddb.jp/search_organism.cgi’. These entries include (i) scientific name, (ii) lineage, (iii) carotenoid profile and (iv) reference list describing the carotenoid profiles. The details are described in Table 2.

**Table 2.** The data content of source organism entries (December 2016 release)

Field	Data content
NAME	Scientific name of source organism
NCBI taxonomy ID	Taxonomy ID defined by NCBI
LINEAGE	Full lineage defined by NCBI
DESCRIPTION	Popular names and explanations of source organism
CAROTENOID PROFILE	List of CA-numbers, structures, descriptions of the carotenoids and reference numbers
REFERENCES	References describing the carotenoid profiles of the source organism

The lineage in all levels is linked to relevant lists of carotenoids. For example, in the carotenoid profile of a cyanobacterium ‘Nostoc commune’ at ‘http://carotenoiddb.jp/ORGANISMS/Nostoc_commune.html’, the list of all carotenoids in the Nostoc genus is available at ‘http://carotenoiddb.jp/ORGANISMS/Nostoc.html’, and a list of all carotenoids in the Nostocaceae family is available at ‘http://carotenoiddb.jp/ORGANISMS/Nostocaceae.html’. 

**Figure 2.**
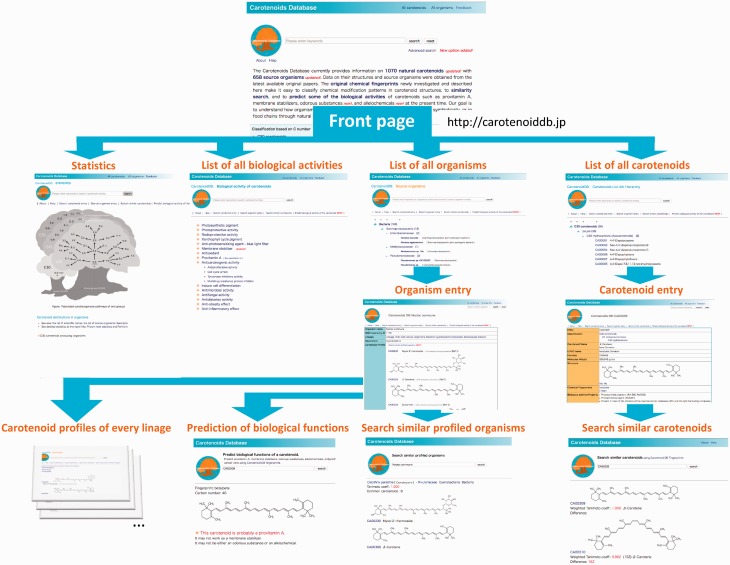
Links in the Carotenoids Database.

Links from the front page of the Carotenoids database are shown in Figure 2.

### Data sources

**Figure 3.**
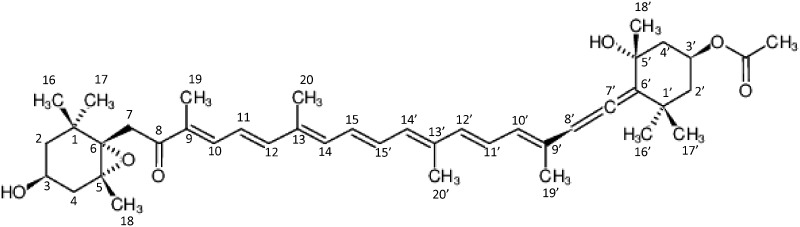
Fucoxanthin: (3S,5R,6S,3'S,5'R,6'R)-5,6-Epoxy-3'-ethanoyloxy-3,5'-dihydroxy-6',7'-didehydro-5,6,7,8,5',6'-hexahydro-beta,beta-caroten-8-on whose chemical fingerprints are made as “(3S,5R,6S,3'S,5'R,6'R), 6',7'–H, 5,6 + H, 7,8 + H, 5',6'+H, 3-OH, 5'-OH, 3'-Ethanoyloxy, 8 = O, 5,6-Epoxy, beta,beta”.

Information on carotenoid structures, biological functions and source organisms has been collected from the latest available original papers, reviews, and books via Google scholar, PubMed systems and Chemical Abstract Service. We also refer and link to other databases such as the KEGG COMPOUND database (12), the KNApSAcK database (13), the Lipidbank database (14) and the ProCarDB (15). If no IUPAC semi-systematic names were shown in the source articles, we supplied one. Based on IUPAC semi-systematic names, we define Carotenoid DB Chemical Fingerprints. Chemical structures are hand drawn with the chemical drawing tools Marvinsketch (https://www.chemaxon.com/products/marvin/marvinsketch/) and KegDraw (http://www.kegg.jp/kegg/download/kegtools.html). We use Open Babel (http://openbabel.org/wiki/Main_Page) to generate InChI, InChIKey and Canonical SMILES. We calculate molecular weights based on Standard Atomic Weights 2015, defined by the Chemical Society of Japan. We make visual counts of numbers of conjugated double bonds and multiple bonds. Structural isomers and stereoisomers, such as *cis*/*trans* isomers, conformers and enantiomers are extracted by Carotenoid DB Chemical Fingerprints and chemical formula. Chemical values of XLogP, hydrogen bond donors, hydrogen bond acceptors by Lipinski's definition, Lipinski Failures, complexity of molecule, number of heavy atoms and topological polar surface area are calculated by the PaDEL-Descriptor (16). Carotenoid DB Chemical Fingerprints and conventional fingerprints are downloadable at http://carotenoiddb.jp/FTP/. 12 conventional fingerprints including Pubchem fingerprint (ftp://ftp.ncbi.nlm.nih.gov/pubchem/specifications/pubchem_fingerprints.txt), KlekotaRoth fingerprint (17) and Estate fingerprint (18) are generated by the PaDEL-Descriptor (16) (Figure 3).

We basically make monthly updates as declared in the release notes. See: http://carotenoiddb.jp/releasenotes_2016.html.

## Carotenoid DB Chemical Fingerprints

The ‘Carotenoid DB Chemical Fingerprints’ which we have newly created here are produced using the IUPAC semi-systematic names. The IUPAC semi-systematic names are well-assigned following the Nomenclature of Carotenoids approved by the IUPAC and the IUPAC-IUB Commissions (11). We give here an example of how to produce the fingerprint of the algal carotenoid fucoxanthin (11), for which the IUPAC semi-systematic name is ‘(3S,5R,6S,3′S,5′R,6′R)-5,6-Epoxy-3′-ethanoyloxy-3,5′-dihydroxy-6′,7′-didehydro-5,6,7,8,5′,6′-hexahydro-beta,beta-caroten-8-one’. First, we split the string with hyphens plus numbers. Second, we rewrite ‘3,5’-dihydroxy’ as ‘3-OH, 5′-OH’, ‘6′,7′-didehydro’ as ‘6′,7′-H’, ‘5,6,7,8,5′,6′-hexahydro’ as ‘5,6 + H, 7,8 + H, 5′,6′+H’, ‘8-one’ as ‘8 = O’. Finally, the fingerprint for fucoxanthin is obtained as ‘(3S,5R,6S,3′S,5′R,6′R), 6′,7′–H, 5,6 + H, 7,8 + H, 5′,6′+H, 3-OH, 5′-OH, 3′-Ethanoyloxy, 8 = O, 5,6-Epoxy, beta,beta’. The number in the fingerprints indicates the position-number of the chemically modified carbon atom. These position numbers follow the Nomenclature of Carotenoids approved by the IUPAC and IUB commissions (11).

These fingerprints are described in all possible entries and linked to a category homepage. For instance, ‘3-OH’ with a β, β end group is linked to ‘http://carotenoiddb.jp/FINGERPRINT/2.1_beta,beta_+_3-OH.html’. Fingerprints can be categorized into 23 chemical modification patterns, namely, hydroxylation, saturation, cyclization of end groups, ketolation, desaturation, stereoisomer (RS), apo (cleavage of polyene chain), epoxidation, esterification, *cis*/*trans* isomerization, glycosidation, aldehyde-addition, alkoxylation, carbonylation, isoprene polymerizations, nor (elimination of CH3, CH2 or CH group), complex polymerization, olide formation, sulfation, seco formation (fission of the bond between two adjacent carbon atoms with addition of one or more hydrogen atoms at each terminal group), retro (a shift of all single and double bonds of the conjugated polyene chain), cycloaddition and geranylgeranyl polymerization in the descending order of frequency to our current statistics at the release of December 2016. In other words, every chemical modification pattern in carotenoids is expected to belong to one of those 23 categories in the present investigations. All fingerprints and their detailed descriptions including the definitions of end groups are listed at ‘http://carotenoiddb.jp/Entries/Carotenoid_DB_Chemical_Fingerprint_Help.pdf’. Statistics of Carotenoid DB Chemical Fingerprints are available at ‘http://carotenoiddb.jp/stats/statistics.html’.

## Similarity search with Carotenoid DB Chemical Fingerprints

Using the Carotenoid DB Chemical Fingerprints, we developed a simple scoring method for similarity searches. Similarity searches are possible from each entry, for example for β-carotene at ‘http://carotenoiddb.jp/search_similar_carotenoid.cgi?keyword=CA00309’. In order to evaluate reaction likeliness by the frequency of fingerprints, aside from the Michaelis constant *K*_m_ and/or maximum reaction velocity *V*_max_ values, we introduced weighted Tanimoto coefficient as follows.

Here we define similarity as reaction likeliness:

$$Weighted\;\;Tan\;imoto\;coefficient\;\left(Q,\;E\right)=\frac{W\left(Q\cap E\right)}{W\left(Q\cup E\right)}$$

The fingerprints in every category vary in number of atoms, so we weighted each category of fingerprints to give weighted Tanimoto coefficient (19), inversely proportional to the occurrence rate, with a few exceptions. For example, hydroxylation and saturation occur quite frequently in carotenoids, so we assigned a small weight to those fingerprints.

By this combination of fingerprints and weighted Tanimoto coefficient, we obtained more precise results than with conventional fingerprints in the chemical space of carotenoids within short computational times. Comparisons with other, conventional fingerprints were done by calculating Tanimoto coefficients of all to all pairs of Carotenoids DB entries. See: http://carotenoiddb.jp/FTP/and http://carotenoiddb.jp/FTP/Tanimoto_coeff_eq_1/. All the conventional fingerprints were generated by the PaDEL-Descriptor (http://padel.nus.edu.sg/software/padeldescriptor/).

## Search similar profiled organisms

We have compiled 683 source organisms’ carotenoid profiles. Using these profiles, we have developed a comparison tool. We introduced unweighted Tanimoto coefficients as similarity scores. This ‘Search similar profiled organisms’ is available at ‘http://carotenoiddb.jp/search_similar_profiled_organisms.cgi’.

It seems that we succeeded in extracting species and/or organisms potentially related in some manner to each query organism.

For example, calculating the Tanimoto coefficients for two carotenoid profiles of *Cyanidioschyzon merolae* (20) and *Prochlorothrix hollandica strain PCC 9006* (21) gives unity. That is, both species have the same simple carotenoid profile: β-carotene and zeaxanthin, which is called ZEA-type by Takaichi *et al.* (22). See the profile comparisons at http://carotenoiddb.jp/search_similar_profiled_organisms.cgi?keyword=Cyanidioschyzon%20merolae. The same profile can be found in two other glaucophytes: *Cyanophora paradoxa* (23) and *Glaucocystis nostochinearum* (23) at the same URL. These facts may suggest that the chloroplasts of these primitive unicellular organisms, *Cyanidioschyzon merolae*, *Cyanophora paradoxa* and *Glaucocystis nostochinearum*, may have been derived from the same cyanobacteria which is closely related to *Prochlorothrix hollandica* in agreement with Takaichi *et al.* (22) and Tomitani *et al.* (24).

However, these results are heavily dependent on the conditions, the accuracies, and the fullness of the data found in the original papers. Similarity searching of carotenoid profiles in every lineage is also possible at ‘http://carotenoiddb.jp/search_simiar_profiles_in_all_levels.cgi’, which is linked at every webpage of all lineages. (See ‘http://carotenoiddb.jp/ORGANISMS/Prochlorothrix.html’, for instance).

## Predicting biological functions using Carotenoid DB Chemical Fingerprints

We have also investigated a simple method of predicting six biological functions of carotenoids using Carotenoid DB Chemical Fingerprints, which are as provitamin A, membrane stabilizers, odorous substances, allelochemicals, antiproliferative activity and reverse MDR activity against cancer cells. Feature extractions are based on empirical findings from the latest original papers, which are listed at ‘http://carotenoiddb.jp/Biological_activity/biological_activities_list.html’. Chemically unmodified carotenoids with β end groups can be expected to be provitamin A. Carotenoids with oxygen on both end groups are potentially membrane stabilizers. Namely, fingerprints including oxygen, such as ‘=O’ describing ketone, ‘-Methoxy’ describing methoxy, ‘-Epoxy’ describing epoxy, ‘-Glc’ describing glucoside, ‘-al’ describing aldehyde, ‘-SO4’ describing sulfate with carbon-numbering with and without prime, indicating both ends of the carbons are potentially membrane stabilizers. Carotenoids whose carbon number is < 20 with oxygen attached such as ketone *‘*=O’, hydroxylation ‘-OH’, aldehyde ‘-al’, epoxydation ‘-Epoxy’ can be expected to be odorous substances, and/or allelochemicals. Carotenoids with epoxidized β end groups with β ends on the other side such as Fucoxanthin and Peridinin function as antiproliferative agents against cancer cells (25). Therefore, carotenoids with fingerprint ‘5,6-Epoxy’ or ‘5,8-Epoxy’ with and/or without prime, and ‘beta,beta’ are predicted as possible antiproliferative agents against cancer cells. Likewise, epoxycarotenoids having β,β or β,κ or β,ε end groups (Capsochrome, for example) function as reverse MDR agents against cancer cells (25). That is, carotenoids with fingerprints ‘5,6-Epoxy’ and/or ‘5,8-Epoxy’ with and/or without prime, and ‘beta,beta’ or ‘beta,kappa’ or ‘beta,epsilon’ are potentially reverse MDR agents.

## Classification of carotenoids using Carotenoid DB Chemical Fingerprints

Carotenoids are classified along with their biosynthesis pathways. We simplified them into three steps; first, by carbon numbers: C30, C40, C45 and C50 carotenoids, second, by end-groups, of which there are seven: ψ, β, γ, ε, φ, χ and κ, and third, by chemical modification pattern, that is, hydrocarbons, hydroxycarotenoids, epoxycarotenoids, aldehydes, ketones, carboxylic acids, apocarotenoids, norcarotenoids, secocarotenoids, retrocarotenoids, olidecarotenoids, allenecarotenoids, acetylenecarotenoids and diapocarotenoids. Carotenoid DB Chemical Fingerprints allowed easier classification as shown in Table 3. Bold characters are the necessary fingerprint of each carotenoid. All these types of carotenoids are available from the links in the front page of ‘http://carotenoiddb.jp’.

**Table 3.** Category of carotenoids and including Carotenoid DB Chemical Fingerprints

Carotenoids category	Categories of including Carotenoid DB Chemical Fingerprints	Examples of entries, and their fingerprints		
**Hydrocarbons**	**End groups,** and/or *cis*/*trans*, and/or saturation/desaturation	CA00047	Neurosporene	7,8+H, **psi,psi**
**Hydroxycarotenoids**	End groups, and/or *cis*/*trans*, and/or saturation/desaturation and glycosylation/**hydroxylation**/alkoxylation	CA00322	β-Cryptoxanthin	(3R), 3-**OH**, beta,beta
**Epoxycarotenoids**	End groups, and/or *cis*/*trans*, and/or saturation/desaturation, and/or glycosylation/hydroxylation/alkoxylation and **epoxydation**	CA00628	α-Carotene epoxide	5,6+H, 5,6-**Epoxy**, beta,epsilon
**Aldehydes**	End groups, and/or *cis*/*trans*, and/or saturation/desaturation, and/or glycosylation/hydroxylation/alkoxylation and/or epoxydation, and **aldehyde addition**	CA00161	Anhydrorhodovibrinal	3,4–H, 1,2+H, 1-Methoxy, 20-**al**, psi,psi
**Ketones**	End groups, and/or *cis*/*trans*, and/or saturation/desaturation, and/or glycosylation/hydroxylation/alkoxylation, and/or epoxydation, and/or aldehyde addition and **ketolation**	CA00184	Ketohydroxylycopene	3′-OH, 4**=O**, psi,psi
**Carboxylic acids**	End groups, and/or *cis*/*trans*, and/or saturation/desaturation, and/or glycosylation/hydroxylation/alkoxylation, and/or epoxydation, and/or aldehyde addition and/or ketolation, and **carbonylation/olide**	CA00283	Torularhodin	3′,4′–H, 16′-**COOH**, beta,psi
**Apocarotenoids**	End groups, and/or *cis*/*trans*, and/or saturation/desaturation, and/or glycosylation/hydroxylation/alkoxylation, and/or epoxydation, and/or aldehyde, and/or ketolation, and/or carbonylation/olide, and/or nor and **apo**	CA00288	Neurosporaxanthin	4′-COOH, 4′-**apo**, beta,psi
**Norcarotenoids**	End groups, and/or *cis*/*trans*, and/or saturation/desaturation, and/or glycosylation/hydroxylation/alkoxylation, and/or epoxydation, and/or aldehyde, and/or ketolation, and/or carbonylation/olide, apo and **nor**	CA00572	Actinioerythrol	(3S,3’S), 3-OH, 3′-OH, 4=O, 4′=O, 2-**nor,**2′-**nor**, beta,beta
**Secocarotenoids**	End groups, and/or *cis*/*trans*, and/or saturation/desaturation, and/or glycosylation/hydroxylation/alkoxylation, and/or epoxydation, and/or aldehyde, and/or ketolation, and/or carbonylation/olide and **seco**	CA00584	β-Carotenone	5=O, 6=O, 5′=O, 6′=O, 5,6-**seco**, 5′,6′-**seco**,beta,beta
**Retrocarotenoids**	End groups, and/or *cis*/*trans*, and/or saturation/desaturation, and/or glycosylation/hydroxylation/alkoxylationy, and/or epoxydation, and/or aldehyde, and/or ketolation, and/or carbonylation/olide, and/or apo and **retro**	CA00196	Retrodehydro-γ-carotene	4′,5′–H, 4,5′-**retro**, beta,psi
**Olidecarotenoids**	End groups, and/or *cis*/*trans*, and/or saturation/desaturation, and/or glycosylation/hydroxylation/alkoxylation, and/or epoxydation, and/or aldehyde, and/or ketolation, and/or carbonylation/olide, and/or nor and **olide**	CA00413	Peridininol 5,8-furanooxide	(3S,5R,6S,3’S,5’R,6’S), 6′,7′–H, 5,6+H,5′,6′+H, 3-OH, 3′-OH, 5’-OH, 5,8-Epoxy,19,11-**olide**, beta,beta
**Allenecaroetenoids**	End groups, and/or *cis*/*trans*, and saturation/**desaturation (6,7–H)**, and/or glycosylation/hydroxylation/alkoxylation, and/or epoxydation, and/or aldehyde, and/or ketolation, and/or carbonylation/olide, and/or nor and/or apo	CA00341	Trollein	(3S,5R,6R,3’R), **6,7–H**, 5,6+H, 3-OH,5-OH, 3′-OH, beta,beta
**Acetylenecarotenoids**	End groups, and/or *cis*/*trans*, and saturation/**desaturation (7,8–H)**, and/or glycosylation/hydroxylation/alkoxylation, and/or epoxydation, and/or aldehyde, and/or ketolation, and/or carbonylation/olide, and/or nor and/or apo	CA00296	Crassostreaxanthin A	(3R,3’R,5’R,6’S), **7,8–H**, 1′,2′+H, 5′,6′+H,7′,8′+H, 3-OH, 1′=O, 8′=O, 3′,6′-Epoxy,16′-nor, beta,psi
**Diapocarotenoids**	End groups, and/or *cis*/*trans*, and saturation/desaturation, and/or glycosylation/hydroxylation/alkoxylation, and/or epoxydation, and/or aldehyde, and/or ketolation, and/or carbonylation/olide, and/or nor and **two apos**	CA00886	Crocetindial	8-al, 8′-al, 8**-apo**, 8′**-apo**

## Statistics

### Distribution among organisms

Based on the facts that β, γ and ε rings are formed from ψ ends, and φ, χ and κ rings are formed from β end groups (1), we can postulate that the carotenogenesis pathways may have evolved dendritically (Table 4). The phyla of source organisms producing each end group with carbon numbers are listed in Table 5. Updated lists are also available at ‘http://carotenoiddb.jp/stats/stats_endgroup_phylums.html’, as well as the lists of scientific names of organisms at ‘http://carotenoiddb.jp/stats/stats_endgroup_org_detailed.html’, and the lists of families of organisms at ‘http://carotenoiddb.jp/stats/stats_endgroup_family.html’.

**Table 4.** Numbers of carotenoids in the three domains of life (December 2016 release)

Domains of life	Number of organisms	C30 carotenoids	C40 originated carotenoids	C45 carotenoids	C50 carotenoids	Total number of carotenoids
Archaea	8	1	14	0	5	19
Bacteria	170	33	243	7	24	307
Eukaryotes	505	0	607	0	0	607

**Table 5.** Distribution of carbon numbers and end groups of carotenoids among organisms at phylum level (December 2016 release)

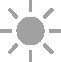 C30 carotenoid producing organisms	
End groups	Source organisms
ψ,ψ	Euryarchaeota, Firmicutes, Cyanobacteria, Alphaproteobacteria, Gammaproteobacteria
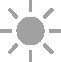 C40 originated carotenoid producing organisms	
End groups	Source organisms
ψ,ψ	Cyanobacteria, Deinococci, Alphaproteobacteria, Betaproteobacteria, Deltaproteobacteria, Gammaproteobacteria, Actinobacteria, Firmicutes, Gemmatimonadetes, Cryptophyta, Streptophyta, Chordata, Chlorophyta, Basidiomycota, Ascomycota, Porifera, Arthropoda (Insecta)
β,ψ	Cyanobacteria, Actinobacteria, Deinococci, Alphaproteobacteria, Gammaproteobacteria, Bacteroidetes, Chlorobi, Firmicutes, Deltaproteobacteria, Euglenida, Chlorophyta, Streptophyta, Basidiomycota, Ascomycota, Chordata, Porifera, Mollusca, Arthropoda (Insecta)
ε,ψ	Streptophyta, Chordata
γ,ψ	Arthropoda (Insecta)
φ,ψ	Gammaproteobacteria, Unclassified bacteria (Chlorochromatium), Chlorobi, Actinobacteria
χ,ψ	Gammaproteobacteria
β,β	Crenarchaeota, Euryarchaeota, Cyanobacteria, Deinococci, Alphaproteobacteria, Actinobacteria, Bacteroidetes, Rhodophyta, Chlorophyta, Streptophyta, Cryptophyta, Eustigmatophyceae, (Stramenopiles), Haptophyceae, Alveolata, (Raphidophyceae), Bacillariophyta, Euglenida, Unclassified chlorophyta, Phaeophyceae, Glaucocystophyceae, Basidiomycota, Porifera, Arthropoda, Mollusca, Ascomycota, Chordata, Echinodermata, Cnidaria, Arthropoda (Insecta)
β,ε	Euryarchaeota, Cyanobacteria, Rhodophyta, Chlorophyta, Streptophyta, Cryptophyta, Haptophyceae, Euglenida, Unclassified chlorophyta, Mollusca, Chordata, Porifera, Ascomycota, Echinodermata, Actinobacteria, Arthropoda, Cnidaria, Arthropoda (Insecta)
β,γ	Ascomycota, Mollusca, Streptophyta, Arthropoda (Insecta)
ε,ε	Cyanobacteria, Cryptophyta, Chlorophyta, (Stramenopiles), Mollusca, Streptophyta, Chordata
γ,ε	Chlorophyta, Unclassified chlorophyta, Porifera
γ,γ	Arthropoda (Insecta)
β,φ	Gammaproteobacteria, Porifera
β,χ	Gammaproteobacteria, Echinodermata, Porifera
β,κ	Streptophyta, Chordata, Mollusca, Porifera, Echinodermata, Ascomycota
κ,χ	Porifera
κ,κ	Streptophyta
φ,φ	Actinobacteria, Gammaproteobacteria, Chlorobi, Unclassified bacteria (Chlorochromatium), Mollusca, Porifera
χ,χ	Cyanobacteria, Gammaproteobacteria
φ,χ	Gammaproteobacteria, Porifera
ψ,-	Cyanobacteria, Streptophyta, Ascomycota
β,-	Streptophyta, Haptophyceae, Mollusca, Cyanobacteria, Gammaproteobacteria, Alphaproteobacteria, Chordata, Actinobacteria, Ascomycota, Echinodermata, Bacillariophyta, Arthropoda (Insecta), Porifera, Arthropoda
ε,-	Streptophyta, Mollusca, Chordata, Ascomycota, Arthropoda (Insecta)
γ,-	Streptophyta
κ,-	Streptophyta
no end group	Cyanobacteria, Gammaproteobacteria, Streptophyta, Ascomycota
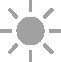 C45 carotenoid producing organisms	
End groups	Source organisms
ψ,ψ	Actinobacteria
β,ψ	Bacteroidetes
ε,ψ	Actinobacteria
β,β	Actinobacteria
ε,ε	Actinobacteria
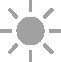 C50 carotenoid producing organisms	
End groups	Source organisms
ψ,ψ	Euryarchaeota, Actinobacteria, Unclassified bacteria (Halophilic bacteria)
β,ψ	Actinobacteria
ε,ψ	Actinobacteria
β,β	Actinobacteria
ε,ε	Actinobacteria, Gammaproteobacteria
γ,γ	Firmicutes, Actinobacteria

Carotenoids are widely distributed in the three domains of life according to our current investigations. Archaea produce C30 ψ, ψ carotenoids, C40 ψ, ψ carotenoids, β, β carotenoids, β, ε carotenoids, C50 ψ, ψ carotenoids and apocarotenoids. Bacteria produce wider ranges of carotenoids, except that they do not produce C40 ε, ψ, C40 γ end or C40 κ end carotenoids. Eukaryotes produce only C40 originated carotenoids, including apocarotenoids numbering 154. Source references are all listed in carotenoid entries and organism entries.

Although the numbers of organisms we could compile are not evenly distributed in the three domains of life (Archaea: 8, Bacteria: 170 and Eukaryotes: 505), and not all the carotenoid entries could be linked to source organisms in the time so far available, our statistics on distribution in organisms show some insights into natural history. Carotenoids seem to have been diversified largely by bacteria, as they produce C30, C40, C45, C50 carotenoids and C40 originated apocarotenoids, with the widest range of end groups, numbering 307. Bacteria share 52 C40 carotenoids and C40 originated apocarotenoids with eukaryotes, and archaea share only seven with eukaryotes. In terms of carotenoids, eukaryotes seem more closely related to bacteria than to archaea, aside from 16S rRNA lineage analysis. This may be caused by the restricted number of reports on archaeal carotenoids (26). Eukaryotes then probably have evolved a considerable number of C40 carotenoids and their derivatives apocarotenoids, numbering 607 by our present count at the release of December 2016.

**Figure 4.**
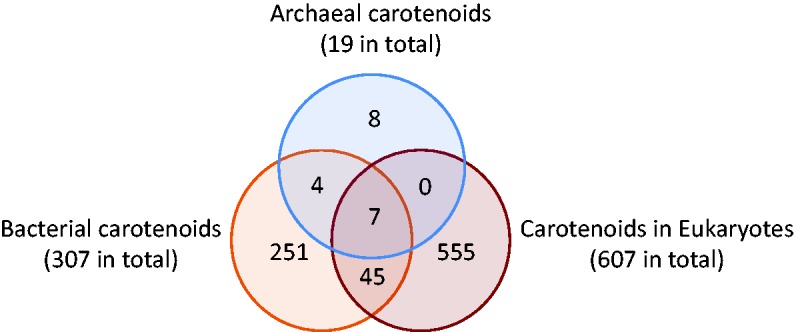
Numbers of unique carotenoids and common carotenoids in the three domains of life. (December 2016 release).

According the data available to us up to the time of the December 2016 release, the common carotenoids in the three domains of life shown in Figure 4 are only hydrocarbons: phytoene, 15-*cis*-phytofluene, all-*trans*-phytofluene, lycopene, β-carotene, (13Z)-β-carotene and α-carotene. (9Z)-β-carotene and lutein were not found in Archaea to our current knowledge. Additionally, bacteria share 45 more carotenoids with eukaryotes: prolycopene, 3,4-dehydrolycopene, bisdehydrolycopene, ζ-carotene, asymmetric ζ-carotene, neurosporene, phillipsiaxanthin, spheroidenone as C40 psi,psi carotenoids, γ-carotene, rubixanthin, 1-hydroxy-1,2-dihydroneurosporene and torulene as C40 beta,psi carotenoids, (9Z)-β-carotene, zeaxanthin, isozeaxanthin, β-cryptoxanthin, isocryptoxanthin, echinenone, 3′-hydroxyechinenone, canthaxanthin, adonirubin, mutatochrome, mutatoxanthin as C40 beta,beta carotenoids, ε-carotene, lutein as C40 epsilon,epsilon carotenoids, and tethyatene as C40 beta,chi carotenoids, isorenieratene as C40 phi,phi carotenoid, renieratene as C40 phi, chi carotenoid and 17 apocarotenoids originated from C40 carotenoids: crocetindial, retinal, apo-8′-lycopenal, β-apo-13-carotenone, β-apo-14′-carotenal, β-apo-10′- carotenal, apo-13-zeaxanthinone, apo-15-zeaxanthinal, apo-12′-zeaxanthinal, apo-10′-zeaxanthinal, β-apo-8′- carotenal, β-citraurin, β-citraurinol, β-ionone, 4-oxo-β-ionone, (3R)-3-hydroxy-β-ionone and tectoionols A.

Archaea seem to have originated 10 chemical modifications: hydroxylation, β and ε cyclases, *cis*/*trans* isomerizatin, glycosidation, esterification, saturation, desaturation, epoxidation, apo (cleavage of polyene chain, not yet shown in the database) and isoprene attachment. Bacteria then may have added six chemical modifications, that is, alkoxylation, ketolation, aldehyde attachment, carboxylation, sodium addition, and retro (a shift of all single and double bonds of the conjugated polyene chain). Finally, eukaryotes probably have evolved six chemical modifications and reduced one chemical modification. Seco (fission of the bond between two adjacent carbon atoms with addition of one or more hydrogen atoms at each terminal group), cyclo addition, or (elimination of CH3, CH2 or CH group), olide formations, geranylgeranyl polymerizations and complex polymerizations are observable only in eukaryotes, and isoprene attachment has not been found in eukaryotes to our knowledge.

Updated information on the distribution of chemical modification details at phylum level is available at ‘http://carotenoiddb.jp/stats/org_statistics_phylum.html’. Distribution of chemical modifications at family level is also available at ‘http://carotenoiddb.jp/stats/org_statistics_family.html’.

Updated information on common carotenoids and unique carotenoids in the three domains of life are available at http://carotenoiddb.jp/ORGANISMS/common_carotenoids.html.

## Summary and future works

We have developed the Carotenoids Database to provide chemical information on 1117 natural carotenoids with 683 source organisms. Our newly developed Carotenoid DB Chemical Fingerprints make classification easier and similarity searching precise among carotenoids known to us. Also, the Carotenoid DB Chemical Fingerprints have made it easy to predict six biological functions of carotenoids, that is (i) provitamin A, (ii) membrane stabilizers, (iii) odorous substances, (iv) allelochemicals, (v) antiproliferative activity against cancer cells and (vi) reverse MDR activities. We have newly developed a tool to search for similar profiled organisms, that helps extracting organisms potentially closely related to any query organism, evolutionarily, or symbiotically, or in food chains. Although the numbers of organisms that we have been able to include so far are not evenly distributed in the three domains of life, our statistics on distributions among organisms give some insights into natural history. Carotenoids seem to have been diversified largely by bacteria. Bacteria and archaea seem to have shared small portions of C40 carotenoids with eukaryotes. Eukaryotes then probably have evolved a considerable number of C40 carotenoids. In terms of carotenoids, eukaryotes seem more closely related to bacteria than to archaea, aside from 16S rRNA lineage analysis. In our current investigation, seco, nor, cyclo, olide carotenoids, geranylgeranyl and complex structure polymerized carotenoids are only observable in eukaryotes. See current statistics at http://carotenoiddb.jp/stats/org_statistics_phylum.html.

To promote understanding of how organisms are related via carotenoids, further development of fingerprints for de novo reconstruction of carotenoid biosynthesis pathways will be reviewed in a later paper.
